# The Impact of Contained Versus Uncontained Morcellation Following Laparoscopic Myomectomy on Operation Time, Pain Perception and Bleeding

**DOI:** 10.1111/jog.70471

**Published:** 2026-08-23

**Authors:** Ann‐Sophie Jensen, Martin Rudnicki

**Affiliations:** ^1^ Department of Obstetrics and Gynecology Odense University Hospital Odense Denmark; ^2^ Department of Clinical Research, Faculty of Health Science University of Southern Denmark Odense Denmark

**Keywords:** gynecologic, laparoscopic, myomectomy, surgery

## Abstract

**Title:**

The impact of contained power versus uncontained manual morcellation following laparoscopic myomectomy on operation time, pain perception, and bleeding.

**Purpose:**

Knowledge of contained power morcellation in relation to operation time, bleeding, and in‐hospital‐stay is sparse. Our study aimed to investigate how contained power versus uncontained manual morcellation following laparoscopic myomectomy influenced operation time and bleeding.

**Methods:**

Women undergoing laparoscopic myomectomy at Odense University Hospital between September 2019 and September 2024 were included. Inclusion criteria were women ≥ 18 years undergoing a laparoscopic myomectomy. The size of the myomas, number of myomas, and demographics did not affect patient eligibility. Exclusion criteria were robot‐assisted or open myomectomy, and malignancy.

**Results:**

In total, 116 women met inclusion criteria and underwent laparoscopic myomectomy. Of these, 74 (63.8%) underwent contained power morcellation and 42 (36.2%) uncontained manual morcellation. The operation time was 118.0 min (±54.07) in the contained group and 114.5 min (±32.53) in the uncontained manual group (*p* = 0.704). Perioperative bleeding was significantly lower in the contained power group versus the uncontained manual group (224.5 mL [±358.6] vs. 521.1 mL [±497.2], *p* = 0.0005). The length of hospitalization did not differ between contained power group and the uncontained manual group (0.57 days [±0.98] vs. 0.69 days [±0.68]; *p* = 0.473). One woman in the uncontained manual group had an intraabdominal abscess, and two women had intraabdominal bleeding, one in the uncontained manual group and one in the contained power group.

**Conclusion:**

Our study demonstrated that contained power morcellation following myomectomy did not increase the operation time. The contained power morcellation was associated with lower perioperative bleeding; however, this finding should be interpreted cautiously because of the retrospective study design and potential residual confounding.

## 1 Introduction

Uterine leiomyoma (myomas or fibroids) are benign tumors that arise from smooth muscle cells of the myometrium and are the most common pelvic tumor in women [1]. The incidence ranges from 4.5% to 68.6% depending on study population and diagnostic methods [2]. The incidence increases with age but decreases after menopause [3]. Furthermore, the incidence is related to ethnicity [3]. Risk factors beyond genetics include central obesity, insulin resistance, elevated blood pressure, and hyperlipidemia [4, 5]. Myomas are associated with symptoms like heavy menstrual bleeding, pelvic pain, and infertility [6, 7]. Other symptoms are urinary problems, constipation and/or tenesmus, and pelvic pressure [6, 8].

In order to preserve fertility, myomectomy has been the surgical procedure of choice to preserve the uterus [9]. Furthermore, it is crucial for preserving fertility to maintain the endometrial cavity [10]. The surgical methods include laparoscopic myomectomy, open abdominal myomectomy, or vaginal myomectomy. Because of the short postoperative hospitalization and fast recovery related to laparoscopic myomectomy, this procedure is currently the preferred surgical method [11].

One major problem is the extraction of the myomas following myomectomy. Due to the risk of malignancy in 1 in 225 to 1 in 580 among women undergoing surgery for uterine myoma, the US Food and Drug Administration (FDA) advised that laparoscopic power morcellators in women with myomas should only be conducted with a tissue containment system. This is due to the risk of disseminating unsuspected malignancies within the abdominal cavity, which may significantly worsen patient outcomes [12]. Consequently, the FDA recommends that power morcellation, if performed, should only be conducted using an approved tissue containment system. Despite these recommendations, clinical practice varies considerably, as some centers continue to perform robotic or laparoscopic myomectomies without morcellation, whereas others have implemented contained morcellation techniques or alternative specimen extraction methods [13]. This variability in surgical practice highlights the continued importance of contained morcellation as a strategy to balance the benefits of minimally invasive surgery with oncologic safety. Recently, a Cochrane review published in 2020 stated that only sparse data on the effectiveness and safety of in‐bag morcellation at the time of laparoscopic myomectomy are available compared with uncontained power morcellation [13].

Extracorporeal C‐Incision Tissue Extraction (ExCITE) techniques are an alternative to contained morcellation. Della Corte et al. found that using the technique was associated with significantly shorter operation time compared with mini laparotomy [14].

The primary aim of this retrospective study was to investigate whether contained power compared with uncontained manual morcellation increased perioperative bleeding and operation time. Secondary, to investigate if the use of contained power morcellation increased in‐hospital‐stay.

## 2 Methods

The study was performed as a single center study where we retrospectively identified women undergoing laparoscopic myomectomy at the Department of Obstetrics and Gynecology, Odense University Hospital (OUH), Denmark from September 2019 to September 2024. The women were identified using ICD‐10 codes, including KLCB10 and KLCB11. Inclusion criteria were women ≥ 18 years with a laparoscopic myomectomy. The size of the myomas, number of myomas, and demographics did not affect patient eligibility. Exclusion criteria were robot‐assisted or open myomectomy, and case of malignancy.

Information was obtained from the women's medical records. Demographic data included age at surgery, body mass index (BMI), smoking history, comorbidities, and prior abdominal surgery. In addition, information regarding surgical procedure, indication of surgery, and perioperative characteristics of the myomas including size, number, weight of myoma, and operation time were collected. The operation time is the total amount of minutes from skin incision to skin closure. Last, intraoperative and postoperative characteristics and complications, and length of hospitalization were registered.

No case received preoperative GnRH treatment to reduce the size of the myoma. Laparoscopic myomectomy was performed under general anesthesia and the patient placed in 20° Trendelenburg position. Pneumoperitoneum with a maximum at 12 mmHg and CO_2_ was used for insufflation. A 12‐mm trocar was inserted through the umbilicus as the primary access port. Two 5 mm trocars were placed in the left abdomen: one in the left lower quadrant and one in the left upper quadrant, aligned to provide optimal triangulation toward the operative field. An additional 5‐mm trocar was positioned in the right lower quadrant for assistance and instrumentation. The Pneumoliner Containment Extraction System and Moresolution Laparoscopic Power Morcellator from Olympus were used in the power morcellation group. The endobag was placed through the umbilicus and a 5‐mm camera was introduced through the Pneumoliner system together with the power morcellator (single port technology) for removal of the myoma(s). VCare was used for uterine manipulation in all patients [15].

Myomectomy was performed after injection of Vasopressin solution prepared by mixing 20 IU of Vasopressin with 100 mL of saline water to make a total of 100 mL (0.2 IU/mL). In all cases, 40 mL was injected subserous above the myoma followed by dissection and enucleation of the myoma. The incision was closed by V‐Loc using a two‐ or three‐layered technique depending on the defect following myomectomy. The myoma(s) were placed in a contained system with an endoscopic bag or not depending on the surgeon's preference. In our institution, there is no mandatory standard deciding the use of contained versus noncontained morcellation. The choice of technique was therefore determined by the surgeon, based on factors such as myoma size, location, patient characteristics, and intraoperative considerations.

The myoma(s) was removed by either electric power morcellation (Moresolution Laparoscopic Power Morcellator) in the bag using an umbilical incision of 2–3 cm (contained power), or by the ExCITE technique without a bag (uncontained manual), which involves extracorporeal tissue extraction through a protected suprapubic incision using an Alexis retractor system [16]. The surgeons were allowed to enlarge the incision both umbilical and suprapubic if there was a problem with removal of either the bag and its content or the myoma itself. Operating surgeons were experienced in the procedures, and the selection of the procedure was chosen in each case by the surgeon. All surgeons were experienced laparoscopic gynecologic surgeons. Complications were registered in accordance with the Clavien–Dindo Classification [17].

### 2.1 Ethics Statement

The study was carried out in accordance with the Declaration of Helsinki II. Furthermore, the study was in accordance with the Strengthening the Reporting of Observational Studies in Epidemiology (STROBE) reporting guideline [18]. Approval of the article's research was accepted by the Region of Southern Denmark approval (No. 24/45433). Data were registered and stored in OPEN, Open Patient data Explorative Network, Odense University Hospital, Region of Southern Denmark (OPEN [OP_2265]) [19]. Further, OPEN analysis was used for anonymous data entry.

### 2.2 Statistics

Statistical analyses were conducted using STATA for MAC version 18 BE. Numerical data are presented as means ± standard deviations (SD) and 95% confidence intervals (CI). Categorical data are presented as numbers and percentages. Difference between groups were assessed by Fisher's exact test for categorical data due to the small numbers. For numerical data, difference between groups were assessed by *t*‐test as data were normally distributed. Multiple linear regression adjusting for confounders including age, BMI, adhesiolysis, and smoking history was used to assess operation time, perioperative bleeding, and length of hospitalization. Assumptions of multiple linear regression analysis were evaluated by plots of residuals. The multiple linear regression analysis on perioperative bleeding was done on a logarithmic scale due to the results of the model validation, where a non‐normal distribution occurred. The results were back transformed into median ratios as the outcome is numerical data. Statistical significance was defined as a two‐sided probability value of < 0.05.

### 2.3 Consent

In accordance with Danish legislation, written informed consent was not required because this was a retrospective registry‐based study using existing medical records. All data were anonymized before analysis, and no patient‐identifiable information was included.

## 3 Results

In total, 127 women were identified through the ICD‐10 codes. Of these, 116 met inclusion criteria and were included in the study. In total, 74 (64%) underwent contained power morcellation and 42 (36%) manual morcellation. Exclusion was due to open myomectomy (eight women) and wrong coding (three women). Patient characteristics on women who had laparoscopic myomectomy are presented in Table 1. The mean (SD) age was 36.9 (±6.51) years and 34.5 (±5.31) years in the contained power and uncontained manual group, respectively. Overall, 30 (25.9%) women had prior surgery in the abdomen and 3 (2.6%) women used anticoagulant medicine before surgery, which all had relevant pause before surgery according to standard regulations [20].

**TABLE 1:** Patient demographic on women undergoing laparoscopic myomectomy.

	Contained power morcellation, *N* = 74 (64%)	Uncontained manual morcellation, *N* = 42 (36%)	*p*
Age (year, mean [±SD])	36.9 (±6.51)	34.5 (±5.31)	0.085
BMI (kg/m^2^, mean [±SD])	25.5 (±4.83)	25.0 (±4.31)	0.589
Prior abdominal surgery (*N* [%])	17 (22.9)	13 (31.0)	0.381
Use of anticoagulant medicine (*N* [%])	2 (2.7)	1 (2.4)	> 0.99
Diabetes (*N* [%])	1 (1.4)	1 (2.4)	> 0.99
Thyroid gland disease (*N* [%])	5 (6.8)	1 (2.4)	0.415
Chronic lung disease (*N* [%])	2 (2.7)	1 (2.4)	> 0.99

Table 2 shows the characteristics of surgery on women undergoing laparoscopic myomectomy. The most frequent reason for surgery was infertility (25 [33.8%] vs. 16 [38.1%]) followed by abnormal uterine bleeding (16 [21.6%] vs. 10 [23.8%]). The mean duration of operation time was 118.0 min (±54.07) for contained power morcellation versus 114.5 min (±32.53) for uncontained manual morcellation. The mean number of myomas removed were 2.48 (±2.54) versus 2.36 (±4.84) and the mean size of the largest myoma were 69.12 mm (±27.47) versus 81.86 mm (±35.74).

**TABLE 2:** Surgical characteristics on women undergoing laparoscopic myomectomy.

	Contained power morcellation, *N* = 74 (64%)	Uncontained manual morcellation, *N* = 42 (36%)	*p*
Indication of surgery (*N* [%])			0.576
Abnormal uterine bleeding	16 (21.6)	10 (23.8)	
Pain	8 (10.8)	5 (11.9)	
Infertility	25 (33.8)	16 (38.1)	
Problems related to urination	7 (9.46)	1 (2.4)	
Obstipation	1 (1.35)	0 (0)	
Sensation of heaviness	2 (2.70)	4 (9.5)	
Unknown	5 (6.8)	1 (2.4)	
Other	10 (13.5)	5 (11.9)	
Duration of operation (min, mean [±SD])	118.0 (±54.07)	114.5 (±32.53)	0.704
Size of largest myoma (mm, mean [±SD])	69.12 (±27.47)	81.86 (±35.74)	0.035^a^
Numbers of myoma (mean [±SD])	2.48 (±2.54)	2.36 (±4.84)	0.861
Adhesiolysis (*N* [%])	12 (16.2)	5 (11.9)	0.596
Entry into the uterine cavity, (*N* [%])	12 (16.2)	13 (31.0)	0.099

^a^ Statistically significant.

The mean perioperative bleeding for the groups was 224.5 mL (±358.6) versus 521.1 mL (±497.2) and the mean length of hospitalization was 0.57 days (±0.98) versus 0.69 days (±0.68). In total, 31 (41%) women in the contained power morcellation group had their umbilical incision enlarged, whereas 12 (28%) women in the uncontained manual group underwent a mini‐laparotomy > 5 cm.

Three women underwent reoperation (Table 3). One woman, in the uncontained manual group, because of an intraabdominal abscess and two women because of postsurgical intraabdominal bleeding, one in the uncontained manual group and one in the contained power group. Overall, in relation to the Clavien–Dindo Classification, six women were classified as Grade II because of blood transfusions, and three women were classified as Grade IIIb due to interventions under general anesthesia [21]. Among the six women who received blood transfusions, three were in the contained power group, while the remaining three were in the uncontained manual group. One of the women from the contained power group was reoperated because of intraabdominal bleeding.

**TABLE 3:** Surgical complications and postoperative characteristics on women undergoing laparoscopic myomectomy.

	Contained power morcellation, *N* = 74	Uncontained manual morcellation, *N* = 42	*p*
Perioperative bleeding (mL, mean [±SD])	224.5 (±358.6)	521.1 (±497.2)	0.0005^a^
Length of hospitalization (days, mean [±SD])	0.57 (±0.98)	0.69 (±0.68)	0.473
Readmission (*N* [%])	2 (2.7)	1 (2.4)	> 0.99
Reoperation (*N* [%])	1 (1.35)	2 (4.8)	0.297
Intraabdominal abscess	0 (0)	1 (0.02)	0.362
Intraabdominal bleeding	1 (0.01)	1 (0.02)	1.00
Conversion to open surgery (*N* [%])	0 (0)	0 (0)	
Use of postoperative in hospital painkillers (*N* [%])	64 (86.5)	38 (90.5)	0.592
Paracetamol	46 (62.2)	31 (73.8)	0.226
NSAID	45 (60.8)	27 (64.3)	0.843
Opioids	35 (47.3)	21 (50.0)	0.848
Other	0 (0)	0 (0)	
Unknown	5 (6.8)	4 (9.5)	0.721
VAS pain score on postoperative pain in hospital (mean [±SD])	2.07 (±2.25)	3.08 (±2.50)	0.075
Clavien–Dindo Classification (*N* [%])			0.401
I	70 (94.6)	37 (88.1)	
II	3 (4.1)	3 (7.1)	
IIIa	0 (0)	0 (0)	
IIIb	1 (1.4)	2 (4.8)	
IVa	0 (0)	0 (0)	
IVb	0 (0)	0 (0)	
V	0 (0)	0 (0)	

^a^ Statistically significant.

Multiple linear regression analysis was used to estimate the relationship between multiple variables and the operation time, perioperative bleeding, and length of hospitalization.

Neither contained power morcellation or uncontained manual morcellation techniques were associated with operation time after adjustment for age, BMI, adhesiolysis, and smoking history, whereas myoma size (adjusted coefficient 0.36, 95% CI 0.03–0.68, *p* = 0.03) and number of myomas (4.16, 95% CI 0.83–7.49, *p* = 0.02) remained significant predictors.

Furthermore, contained power morcellation was associated with reduced perioperative bleeding (adjusted coefficient and 95% CI [adjusted for age, BMI, adhesiolysis, and smoking history]; 0.38; 0.22–0.65; *p* < 0.001) and similarly the increased size and number of myomas were associated with increased bleeding (adjusted coefficient and 95% CI: 1.02; 1.01–1.03; *p* < 0.001 and 1.11; 1.04–1.19; *p* = 0.003, respectively).

The two methods had no impact on in‐hospital‐stay which was influenced by the size (adjusted coefficient [adjusted for age, BMI, adhesiolysis, and smoking history] and 95% CI; 0.01; 0.00–0.01; *p* = 0.026) and the number of myomas (0.03; 0.00–0.05; *p* = 0.037).

## 4 Discussion

Performing laparoscopic myomectomies are challenging and especially to remove the myoma from the abdominal cavity in case of large‐sized myomas. As indicated by the recent Cochrane review only sparse information is available regarding comparison of contained laparoscopic morcellation versus the uncontained technique [13]. Our results indicate that the two techniques presented in this study are equal in respect to operation time and we did not observe any impact on in‐hospital‐stay. However, the in‐hospital‐stay was less than 1 day, and any influence of the surgical methods may therefore be limited.

Our use of contained power morcellation during laparoscopic myomectomy was primarily introduced to reduce the risk of intraperitoneal tissue dissemination, particularly in the context of unexpected malignancy. In our study, contained power morcellation did not prolong operative time, supporting previous findings that safety measures can be implemented without compromising surgical efficiency. Although Chang et al. reported longer operative time in cases undergoing myomectomy their study focused on uterine manipulator use rather than tissue containment and cannot be compared directly. Contrary, Della et al. [15] compared the contained morcellation using the ExCITE technique to mini‐laparotomy without contained morcellation for the extraction of myomas. They found a significant shorter operation time using the contained morcellation and ExCITE technique compared with mini‐laparotomy even though the latter did not include placement of the myomas in a bag [14]. In our study, we found no significant impact on operation time when using contained power morcellation for myoma extraction during laparoscopic myomectomy.

Our study revealed a mean operation time of approximately 2 h irrespectively the procedure. Although shorter, our result is comparable to a study by Chang and Ding who found an operation time that were 135.4 (±48.11) min for cases with a uterine manipulator and 163.4 (±73.65) for cases without a uterine manipulator [22]. Compared with Della Corte et al. our operation time was longer [14]. This suggests that the operation probably depends on the skills of the surgeon merely than the size and numbers of myomas.

Previous studies concluded that myoma size and number of myomas were significant factors increasing both operation time and bleeding during the surgery [22, 23, 24]. This is in accordance with our study, where both the myoma size and number of myomas significantly increases operation time and perioperative bleeding. However, the blood loss in our study was high compared with the study by Wu et al. who reported a blood loss on 217.3 mL (±245.9) during laparoscopic myomectomy, Della Corte. et al. reported a 100–115 mL blood loss. Also, Özbaşlı et al. reported 120 mL blood loss during laparoscopic myomectomy [14, 25, 26]. The size and numbers of myomas causing increased bleeding in the uncontained manual group may be due to an enlarged myometrial incision thereby increasing perioperative bleeding.

Furthermore, different locations of the myomas may act to increase bleeding especially if located cervical or deep, indicated by the more frequent entry into the uterine cavity in the uncontained group which in turn indicates more complex surgery and thereby higher risk of bleeding. Also, pneumoperitoneum may have masked bleeding during laparoscopy. During extracorporeal extraction in the uncontained technique, the loss of intraabdominal pressure may allow previously obscured bleeding to become apparent. However, intraabdominal pressure may also decrease during contained power morcellation, as pneumoperitoneum is maintained within the retrieval bag rather than throughout the abdominal cavity during extraction. Thus, the extent to which changes in intraabdominal pressure may have contributed to the observed difference in blood loss between the two techniques remains uncertain.

Although the increased bleeding observed in the uncontained manual group may in part be due to the increased size of myomas, the difference was still present when adjusted for size in the regression analysis.

Because most women included in our study were young healthy individuals, it was not routine to measure pre and postsurgical hemoglobin levels. Therefore, we are not able to comment on the difference in hemoglobin level before and after surgery. Despite a higher blood loss, only six women had postsurgical blood transfusion, and the higher perioperative bleeding did not seem to affect length of in‐hospital‐stay, which was very short. In addition, the number of women who had blood transfusions were equal in the two groups.

The length of hospitalization seems shorter in our study compared with other studies. A recent systematic review, including eight studies with 890 laparoscopies, evaluating the length of hospital stay following laparoscopic myomectomy ranged from 1.5 to 4 days [27]. Wu et al. demonstrated a hospital stay on 5.09 (±0.74) days among 53 women undergoing laparoscopic myomectomy [25]. However, their cohort differed from ours by higher average age of 41.9 years (±6.28). Other parameters like BMI, previous abdominal surgery, number of myomas, and size of myomas were comparable with our study. Thus, the short postoperative in‐hospital‐stay in our study may reflect the low number of complications in our cohort, and that the patients were young healthy women with a low number of comorbidities. Furthermore, the mean VAS‐score postoperative may also contribute to the short hospitalization. When comparing the postoperative pain, our study had a lower VAS pain score postsurgical compared with Wu et al., who found an average VAS pain score that were 4.13 (±1.35) in the laparoscopic group [25]. They also found a higher use of opioids following laparoscopic myomectomy, where 69.8% had opioids compared with 48.3% in our study. Özbaşlı and Güngör reported median maximum VAS score of 5.5 following laparoscopic myomectomy [26]. This inconsistency between studies regarding postoperative pain may be affected by several factors such as the pain assessment methods, administration of local anesthesia around the port sites, and surgeon's technique and experience.

Entry into the uterine cavity during surgery occurred in 21.6% of the surgical procedures in our study. Breaching the uterine cavity during laparoscopic myomectomy has been demonstrated to be associated with obstetric risks during subsequent pregnancies such as uterine ruptures and placenta accreta spectrum [28, 29]. Additionally, a recent study found that the incidence of uterine cavity entered during myomectomy was significantly higher in the group of planned cesarean deliveries compared with women with trial of labor after myomectomy, whereas they did not observe any impact on baby take home rate in case of previously myomectomy [29]. However, our study could not comment on long‐term complications and following pregnancies, as the follow‐up time was short and only related to postoperative findings, but to reduce uterine rupture we perform two‐ or three‐layered closure at our institution. Additionally, the short follow‐up time also precludes any assessment in postoperative parasitic myomas.

One strength of the study is that all women with laparoscopic myomectomies were included, and patient characteristics did not affect the inclusion. The main limitation is that the study is a single center setup, which leads to a low number of patients. Furthermore, women were identified by ICD‐10 codes and even though the codes were selected wisely, we cannot exclude that some women were not identified due to miscoding, leading to a smaller patient population. Due to the small number of women who underwent robot‐assisted myomectomies, the results were excluded for analysis. In addition, we could not comment on the difference in outcomes between laparoscopic and robot‐assisted myomectomy. Furthermore, a limitation of this study is the lack of standardized data on myoma localization (e.g., FIGO classification) and myoma weight in most cases. Both myoma localization and weight may influence both surgical complexity and bleeding. However, incomplete data precluded its inclusion in the multivariate analysis. Last, extraction time was not documented separately in the available records, and we therefore cannot distinguish it from the overall operative time, limiting conclusions regarding the impact of morcellation technique on surgical efficiency.

In conclusion, our results demonstrate that contained power morcellation did not increase operation time. Furthermore, the procedure appeared safe with respect to perioperative bleeding; however, this finding should be interpreted cautiously given potential confounding factors. Additionally, no differences were found regarding in‐hospital‐stay or adverse outcomes. These findings support that contained power morcellation is a safe and noninferior alternative to the uncontained manual morcellation. Further prospective studies are warranted to confirm these observations and explore long‐term outcomes.

## Author Contributions

**Ann‐Sophie Jensen:** investigation, methodology, validation, software, formal analysis, data curation, writing – review and editing, writing – original draft, funding acquisition, conceptualization. **Martin Rudnicki:** supervision, data curation, formal analysis, project administration, methodology, validation, writing – review and editing, conceptualization, resources.

## Disclosure

The manuscript has not been submitted elsewhere or presented neither in abstract form at a conference or as part of a presentation.

## Conflicts of Interest

The authors declare no conflicts of interest.

## Data Availability

Data from studies conducted in Denmark cannot be shared or transferred with other researchers because strict national privacy laws protect personal information and data are thereby restricted to approved researchers within Denmark.
